# Heart Rate as a Non-Invasive Biomarker of Inflammation: Implications for Digital Health

**DOI:** 10.3389/fimmu.2022.930445

**Published:** 2022-06-02

**Authors:** Martin G. Frasch

**Affiliations:** Department of OBGYN and Center on Human Development and Disability (CHDD), University of Washington, Seattle, WA, United States

**Keywords:** heart rate variability (HRV), immune system, immune memory, digital health (eHealth), machine learning and AI

## Introduction

The purpose of this opinion article is to provide a concise evidence synthesis on the subject of non-invasive monitoring of the inflammatory response using heart rate variability (HRV).

The starting point forms the recent study by Magawa et al. (1) In this study the authors validated and extended the initial observations by Durosier et al. and Herry et al. (2, 3) who have demonstrated that HRV metrics track endotoxin-induced acute fetal inflammatory response in real time as measured by a composite index of HRV metrics and IL-6, the key inflammatory cytokine of the fetal inflammatory response. While ovine fetus was used as the preclinical model, this was the first demonstration of longitudinal tracking of an acute subclinical inflammatory response using ECG-derived HRV in general. The developed HRV inflammatory index was later validated in a neonatal piglet model of a sublethal inflammatory response (4).

The novel contributions by Magawa et al. are the demonstration of the HRV monitoring potential at a younger gestational age, also in ovine fetus model, and with a different regime of endotoxin exposure which seeks to recapitulate the protracted course of fetal inflammation as it may happen in human pregnancy. While any preclinical experimental design remains an approximation of the human physiology, the present independent validation of the ability of HRV to track the acute phase of inflammatory response is an important result.

Perhaps even more important is the confirmatory negative finding that the chronic phase of the fetal inflammatory response does not seem to be reflected in the changes of HRV as analyzed, similar qualitatively to the three days lasting observations by Durosier et al, but expanded considerably by Magawa et al. to a duration of ten days.

## Evidence

There are implications of the current status of evidence to consider that will help to develop the full potential of the HRV monitoring antepartum for early detection and tracking of the inflammatory response.

Beyond fetal health monitoring from the above-mentioned studies, these implications also shed light on the novel directions for the ongoing efforts to uncover the potential of Digital Health approaches leveraging ubiquitous wearables to track the inflammatory response, e.g. to COVID-19, in other ages, from children to adults (5–7).

Notably, in addition to relating the HRV metrics to pro-inflammatory cytokines, such as IL-6, another emergent class of molecular biomarkers of interest is microRNA and long non-coding NRA (lncRNA) (8–12).

First, implicit in the present work is the requirement for an electrocardiogram (ECG) signal which, in fetuses, can be obtained clinically from transabdominal ECG devices with several now commercially available around the world. This is not trivial to keep in mind, as this technology remains not widely available, albeit the acceptance by the mothers appears to be very broad (13). Also, in pediatric and adult heart rate monitors, the most widely used biosensor is a photoplethysmograph (PPG) which poses similar limitations on the quality of HRV data as to what components of HRV code may be captured (14, 15). This will remain a subject of ongoing research.

Second, there remain distinct methodological differences in the approaches to tracking inflammation using HRV. The convention approach remains to focus on the single HRV metrics. In addition, usually, mostly some select conventional linear HRV metrics in time and frequency are considered domain complemented by the a few nonlinear biomarkers of HRV, in the case of Magawa et al, sample entropy and asymmetry, which were also computed as they had performed well in clinical studies of early neonatal sepsis detection (16).

The authors then computed these HRV metrics in predefined hourly averaged intervals, rather than evaluating sample-by-sample dynamics, and did not demonstrate the concomitant changes in the temporal profile of the pro-inflammatory cytokines such as IL-6.

In the case of COVID-19 tracking studies, remarkably, so far even the zero-order metrics of heart rate have been shown to carry promise in detecting differences in immune status as noted above.

This conventional strategy stands in contrast to studies performed with no *a priori* selection of particular HRV metrics, computed continuously, without hourly averaging, and deriving a composite index of inflammatory response, termed the fetal inflammatory index, which comprised a well-defined subset of four HRV metrics deduced *via* non-supervised machine learning approach from the most complete known set of linear and non-linear HRV metrics currently available (2). The HRV metrics stemmed from the energetic domain: multifractal spectrum cumulant of the first order, time irreversibility asymmetry index (notably, similar to the study by Magawa et al.); from the informational domain: Allan factor distance from a Poisson distribution and from the invariant domain: the embedding scaling exponent. Of interest, the fetal heart rate itself was not part of the fetal inflammatory index. Durosier et al. also clearly demonstrated the concomitant tracking of the IL-6 temporal profile in the acute stage of the inflammatory response. In the most recent study by Quer at al., the zero-order property of HRV, resting heart rate, was shown to reflect the reactogenecity to COVID-19 vaccine in a dose-response manner, i.e., it differed with the first and second shots. Such form of immunological memory reflected in resting heart rate indicates the potential of focusing on resting physiological states to better extract the effects of the immune response on the HRV properties.

It seems clear that HRV can track inflammatory response in its acute phase. Therein also lies the limitation of the state of the art: in the clinical setting we cannot choose the phase of the inflammatory response.

The existing evidence takes us to the conclusion that can inform future study designs: preclinical studies agree that the later stage of the fetal inflammatory response (past the first 24h) does not appear to be discernible with the present signal processing techniques based on HRV metrics, whether taken conventionally one by one or using standard machine learning techniques. This remains an important field for future research with clear clinical implications: can we identify fetuses, children or adults who experienced inflammation even after the acute phase, akin to the chronic memory of Zika virus exposure or chronic hypoxia memory demonstrated elsewhere (17, 18). An exciting avenue will also be relating chronic changes in HRV-derived biomarkers with those in miRNA- and lncRNA-based signatures of inflammation.

## Discussion

How can we tackle the challenge of discovering the HRV biomarker of chronic or past inflammation?

To answer this question, it is beneficial to discuss the underlying motivations in the difference of the analytical approaches from the epistemological and the applied physiological points of view. This approach can point to future research directions.

On the one hand there is the unsupervised learning approach which makes no attempt at directly tying any given HRV metric to a physiological behavior. Rather, it assumes the notion of HRV code (14). We can understand the HRV analysis in such experiments to capture the activation of the fetal cholinergic anti-inflammatory pathway (19) by the endotoxin. It has been reported and discussed that while simple HRV metrics like RMSSD may reflect the activity of the cholinergic anti-inflammatory pathway somewhat (20, 21), the complex network activity of such a pathway cannot be captured by any single fHRV metrics; rather, approaches considering the underlying patterns to be akin to HRV code seem to have a higher chance of succeeding in producing predictive models of physiological behavior (14, 22). This is broadly in line with the emerging understanding of the network physiology (23, 24).

On the other hand, there are the approaches following the opposite principle: select *a priori* a subset of HRV metrics believed to be physiologically interpretable or, at least, useful, and evaluate them for the ability to track inflammation. As I discussed elsewhere, there are fundamental, epistemological limitations to such an approach that limit the generalized inference on the predictive utility of HRV considerably (25). Physiological assumptions that certain solitary HRV metrics reflect specific contributions from the branches of the autonomic nervous system have been challenged recently, with RMSSD notably not being affected by complete vagal denervation in fetal sheep near term (22). Moreover, this strategy has failed us so far in producing any clinically actionable HRV monitoring metrics. Those HRV biomarker discovery strategies that did receive FDA approval for clinical use, e.g. for early sepsis detection, have in fact all been the result of unsupervised machine learning approaches followed by dimensionality reduction (16, 26).

Taken together, I propose that adding the higher-order mathematical features of HRV, likely focused on resting states for detection of chronic states of inflammation, carries the potential to boost the performance of such approaches, with some caveat to be considered as to which biosensors are used to record the raw data.

With the above considerations in mind, the inability of certain few HRV metrics to track inflammation singularly, i.e., without any attempt at machine learning on these HRV metrics as model features or addition of other clinically meaningful multivariate continuous or categorical inputs, cannot, by design, rule out the general ability of the HRV approach, including over 60 metrics (27) as well as application of machine learning, possibly deep learning, to detect signature of chronic or past inflammation. This remains an exciting avenue and challenge for future preclinical research and clinical translation, especially in the area of digital health.

## Author Contributions

The author confirms being the sole contributor of this work and has approved it for publication.

## Conflict of Interest

MF holds patents on fetal monitoring, advises Delfina and is the founder and CEO of Vitalink AI.

## Publisher’s Note

All claims expressed in this article are solely those of the authors and do not necessarily represent those of their affiliated organizations, or those of the publisher, the editors and the reviewers. Any product that may be evaluated in this article, or claim that may be made by its manufacturer, is not guaranteed or endorsed by the publisher.
